# Cost analysis of outpatient services for major external structural birth defects: an ingredient approach in selected hospitals in Kiambu County, Kenya

**DOI:** 10.11604/pamj.2024.48.137.40501

**Published:** 2024-07-25

**Authors:** George Nyadimo Agot, Joseph Kibuchi Wang'ombe, Marshal Mutinda Mweu

**Affiliations:** 1 Department of Public and Global Health, Faculty of Health Sciences, University of Nairobi, Nairobi, Kenya

**Keywords:** Major external structural birth defects, cost analysis, outpatient services, ingredient approach, Kenya

## Abstract

**Introduction:**

major external structural birth defects (MESBDs) are known to exert an enormous economic burden on individuals and health services; however, they have been vastly underprioritized as a public health problem in settings where cost analyses are limited. This study aimed at conducting a cost analysis of outpatient services for MESBDs in selected hospitals in Kiambu County, Kenya.

**Methods:**

a cross-sectional descriptive survey was conducted in four hospitals selected for providing outpatient corrective and rehabilitative services to the under-fives. An ingredient approach was used to gather data retrospectively on the cost drivers for castings, bracings, and tendonectomies from healthcare providers' perspectives for a one-year time horizon from January 1^st^, 2018, to December 31^st^, 2018. Prevalence-based morbidity data were extracted from outpatient occupational therapy clinic registers, whereas staff time was gathered through face-to-face inquiries. Associated cost drivers of 349 cases were quantified, valued using prevailing market prices, and categorized as recurrent costs. The unit economic costs were calculated as average costs, expressed in U.S. dollars, and inflated to the U.S. dollar Consumer Price Index from January 2018 to December 2018.

**Results:**

the unit economic cost of all the cases was estimated at $1,139.73; $1,143.51 for neural tube defects (NTDs), $1,143.05 for congenital talipes equinovarus (CTEV), and $1,109.81 for congenital pes planus.

**Conclusion:**

the highest economic burden of MESBDs was associated with NTDs, and CTEV, respectively. We recommend further research to estimate the economic costs of MESBDs among school-going pupils to inform the equitable allocation of resources for health and childhood developmental programs.

## Introduction

Major external structural birth defects (MESBDs) are defined as physical abnormalities of intrauterine origin present from birth, detectable visually, and have significant health and development impacts [1-4]. These defects are potentially fatal and those children who survive beyond infancy require substantial economic resources to deal with lifelong disabilities [5-9]. Worldwide, approximately 134 million births reportedly occur yearly of which 7.9 million (6%) are born with at least a major birth defect, mostly affecting the central and musculoskeletal systems [1-4,10]. Similar observations were made by a study conducted in Kenya in 2019 [11,12]. Although about 3.3 million of these children die before they are five years old, the 3.2 million who survive may be disabled for life if sufficient resources are not dedicated to corrective and rehabilitative health services [1,2,8].

MESBDs continue to occur exerting an enormous economic burden on individuals and health services in developing countries; however, they have been vastly ignored and unappreciated as public health problems attributed to limited estimation of the associated costs [1,2,9,13]. Even though these defects remain a “silent” global public health problem, the highest burden is shouldered by developing countries due to deficient expertise in economic evaluation studies [1-4,6,8,9,14-17]. Hospital charges for newborn children with some form of birth defects have been reported as four to eight times higher than those without any form of defects [15]. Children surviving beyond infancy could require restorative health services to reduce the adverse impacts associated with MESBDs [1,2]. These interventions are described as corrective and rehabilitative outpatient services consisting of castings, bracings, and tendonectomies whose monetary value is referred to as economic costs or opportunity costs [18-24].

The advancements in medical and surgical interventions are known to reduce the severity of lifelong physical disabilities related to MESBDs; however, their costs are catastrophic and prohibitive to many households and public health care systems [1,2,9,17]. Substantial resources are usually allocated to MESBDs within health care systems, yet their costs are seldom estimated due to the rarity and stochasticity of the defects, scantiness of the costing data, inaccurately profiled costing data, and inadequate costing expertise in developing countries [18,25-27]. The scarcity of local epidemiological data and epidemiological study design differences (prevalence/incidence-based) also impede the accuracy of profiled costing data [18,25-27].

Economic costs increase the extent to which health services, individuals, and society are affected by MESBDs because of the forgone benefits of not investing in the next best alternative [15,18-24,28]. Corrective and rehabilitative health care services for MESBDs are critical in reducing the severity of lifelong disabilities and improving the quality of life for the affected children, as well as the economic productivity of the affected families [1,2,9]. Thus, cost analysis is of public health importance in influencing and informing health planning, policy decisions, resource allocations, and assessing health system performance [18-20,22,23,29,30]. Consequently, the objective of this study was to conduct a cost analysis of the outpatient services for MESBDs from the providers' viewpoints using an ingredient approach in selected hospitals in Kiambu County, Kenya.

## Methods

**Study design:** a hospital-based cross-sectional study design was adopted to generate costing data from prevalence-based local morbidity data using an ingredient approach to estimate the economic costs of corrective and rehabilitative outpatient health services from healthcare providers' perspectives. This was the best choice of study design for measuring the unit economic cost of health services as an attribute of the population, and thus provided a snapshot of the burden associated with the ‘silent' public health problem and allowed for generalization of the study results in similar hospital settings in the region. Even though incidence data were readily available and easily accessible for the costing activity, prevalence-based data were excessively preferred to improve the accuracy of the profiled costing data and the estimation of the unit economic costs. This being an observational study adapting cost analysis techniques, it was reported according to STROBE [31] and CHEERS guidelines [32].

**Study settings:** the study was conducted in four hospitals consisting of three county referral hospitals (Kiambu, Thika, and Gatundu), and PCEA Kikuyu orthopedic (faith-based) selected for providing corrective and rehabilitative outpatient health services to children born with MESBDs in the county. The three county referral hospitals were purposively selected being the only public hospitals providing these services in the county, whereas PCEA Kikuyu orthopedic hospital (faith-based) was selected by simple randomization using sealed envelopes between two faith-based hospitals for providing the same services in the county.

**Study population and eligibility criteria of participants:** the study population consisted of all children aged under five years old born to resident women of Kiambu County between January 1^st^, 2014, and December 31^st^, 2018. Cases were defined as live births with at least one clinically obvious major external structural birth defect referenced/or described by assistant occupational therapists and/or orthopedic surgeons and presented to the occupational therapy clinics for care from January 1^st^, 2018, to December 31^st^, 2018. Caregivers of children born with MESBDs were likely to seek outpatient corrective and rehabilitative health services at the study hospitals whether born in or out of the county, thus the eligibility criterion defined above could minimize systemic bias and ensure the reliability of the study results.

**Study perspective, and time horizon:** the data for cost drivers were gathered retrospectively from health care providers' perspective for a one-year time horizon between January 1^st^, 2018, and December 31^st^, 2018 for purposes of maintaining similar currency conversion, and inflation rates.

**Data collection:** before data collection, the Principal Investigator (PI) recruited and trained four nursing graduates as research assistants to ensure that the data abstraction process that spanned from August 1^st^, 2019 to December 30^th^, 2019 was carried out in a standardized manner. We adopted an ingredient approach to gather the prevalence-based caseloads for the costing analysis of corrective and rehabilitative outpatient health services. The cost ingredients of bracing, tendonectomy, and casting interventions quantified consisted of caseloads (morbidity data) by type of MESBDs, the number of braces, the number of bracings, the number of bracing review visits, the number of casting materials, the number of castings, the number of casting review visits, the number of tendonectomies, the number of tendonectomies review visits (recurrent costs), emoluments, staff-time, building space rental, and utility charges (overhead costs). The prevalence-based morbidity data were retrospectively drawn from outpatient occupational therapist registers; described as medical records containing information on health services provided to children with major external structural birth defects. The information captured in these registers included dates of clinic visits, outpatient numbers, names of the patients, patients' age, residences, diagnoses, and therapeutic interventions, among others. Following a predefined inclusion criterion, 349 cases were determined, and associated cost drivers were identified, measured, and valued (quantified) using prevailing market prices and entered into a predetermined secondary data abstraction tool. On the other hand, staff time for the hospitals' executives comprising the medical superintendents, chief nursing officers, orthopedic surgeons, and health administrative officers was gathered through face-to-face inquiries from the occupational therapists being the closest proxies for the officers mentioned above. The ingredient technique and prevalence-based data were chosen for the possibility of generating detailed and improved accuracy of the profiled costing data. The data gathered comprised the following:

**Caseloads/morbidity data:** following a predefined exclusion criterion, 349 cases of MESBDs were considered for the cost analysis.

**Casting:** castings used to stabilize the affected feet consisted of Plaster of Paris (POP) bandages, orthopedic cotton bandages, and glycerine, valued at local market prices. A set of these materials valued at $3.9 were used to cast two cases of club foot. The study computed the number of castings and the number of revisits after the procedure for all cases of CTEV treated using these strategies.

**Braces:** braces consisted of leather foot covers, rubber soles, and metallic rods used to stabilize cases of club feet. Braces were sourced from the local markets as ready-made products, therefore, were valued at $15.31 using prevailing market prices. The study also enumerated the number of braces and the number of revisits after the procedure for all cases of CTEV treated using this strategy.

**The number of tendonectomies:** this was a procedure performed by surgeons to extend the Achilles tendon for club feet. This was largely an outpatient specialized procedure, therefore, existing market hospital charges for outpatient specialized surgical procedures were used to value the cost of tendonectomies estimated at $51.02. Similarly, the number of tendonectomies and the number of revisits after the procedure were computed for all the cases of CTEV that adopted this treatment strategy.

**Emolument for personnel:** the personnel comprised assistant occupational therapists and support staff whose emoluments were estimated based on the respective public service schemes of service for staff with at least ten years of work experience [33-35]. Emoluments for assistant occupational therapists consisted of basic salary, house allowance, commuter allowance, health risk allowance, and health extraneous allowance, whereas, for the support staff comprised basic salary, house allowance, and commuter allowance [33-35]. The monthly salary and benefits for an assistant occupational therapist in “Grade 10” was valued at $1,224.50, whereas support staff in “Grade 14” was valued at $173.50.

**Renting building space:** occupational therapy outpatient clinics were identified within the respective study hospitals whose plinth floor surface areas were measured in square feet and valued based on the existing local market rates for renting building spaces. The total renting space for the four hospitals was estimated at 3,593.63 square feet and valued at $0.37 local market value.

**Utility charges:** utilities included electricity; water and sewerage estimated at $25.51 and $30.61 per month, respectively.

**Supervisory staff-time:** the staff-time for the medical superintends/directors, orthopaedic surgeons, chief nursing officers/directors, and health administrative officers/directors were identified and measured by consensus through face-to-face inquiries made to the assistant occupational therapists being the closest proxies for the above-mentioned officers. The staff time for medical directors and orthopedic surgeons was measured as a single prevailing market price of specialized medical practitioner's consultation valued at $20.40 and quantified for five days a week for one calendar year. The staff time for nursing directors and administrative directors on the other hand was measured and equated to a single prevailing market price of general medical practitioner's consultation valued at $10.20 for five days a week for one calendar year.

**Minimization of biases:** case ascertainment, information, and systemic biases were expected in this study; therefore, the PI began by predefining an eligibility criterion (case definitions) for participation in the study and predetermining secondary data abstraction tool for purposes of reducing case ascertainment biases. On the other hand, information biases were reduced by training the data collectors on secondary data extraction techniques from the outpatient occupational therapy clinics and entering data into the abstraction tools to ensure the process was conducted in a standardized manner. Further, all the registers for the entire one-year study period (2018) were reviewed and listed all the cases of external structural birth defects to reduce ascertainment and information biases in this study. Systemic bias was also reduced by excluding cases of delayed milestones, and/or developmental conditions due to management intervention similarities.

**Statistical analysis:** following data collection, filled secondary data abstraction tools were manually checked daily for accuracy and completeness and subsequently entered in a Microsoft Excel spreadsheet (Microsoft Office Professional Plus 2019) by two independent data managers to reduce potential errors. The PI cross-checked and validated the computerized dataset against predetermined data abstraction tools for analyses. Descriptive qualitative categorical variables were summarized in frequency tables, proportions, and percentages to show their distributions, whereas continuous variables were summarised and presented in means (averages). Costs assigned to the direct cost center consisted of; (i) the names and numbers of specific MESBDs (caseloads/morbidity data), (ii) the number of primary and review visits for castings and/or bracings and/or tendonectomies, (iii) the number of assistant occupational therapists and their emoluments, (iv) the number of support staff and their emoluments, and (v) the floor plinth area for renting. Overheads on the other hand comprised; (i) the number of orthopaedic surgeons and associated staff time, (ii) the number of medical superintendents/directors, and associated staff time, (iii) the number of chief nursing officers/directors and associated staff time, (iv) the number of health administrative officers/directors and associated staff-time, and (v) utility charges for electricity, water, and sewerage. All the costs were categorized as recurrent costs and allocated to the direct cost center whereas overhead costs were shared proportionally among the respective types of MESBDs and allocated to the final costs center for estimation of the economic costs. The unit economic costs were calculated as average costs expressed in U.S Dollars and inflated to the U.S Dollar Consumer Price Index from January 2018 to December 2018 for all types of MESBDs collectively, and individual types of MESBDs. Capital costs did not suffice in this costing study because movable, and fixed capital resources were not considered for valuation. After all, no inventory records existed for furniture, examination couches, and capital donations in kind, whereas motor vehicles, motorcycles, and bicycles were not used either as direct, indirect, or intermediate costs for corrective and rehabilitative health services for the under-fives with MESBDs. The occupational therapy clinics on the other hand also as fixed capital costs were exceedingly small portions of the respective hospitals' floor plinths, hence valued as recurrent costs using prevailing local market prices for building space rental.

**Unit economic costs, currency conversion, and study assumption:** the total (annual) economic costs were calculated for the defects (349 cases) for computing the unit economic costs as an average of the total costs expressed in Kenya Shillings (KES). The unit economic costs were calculated by dividing the total annual costs by the number of cases using the following formula:

$$\text{Unit economic costs }\left(\text{KES}\right)=\frac{\text{Total economic costs}\left(\text{KES}\right)}{\text{Total number of cases}}$$

Further, the unit economic costs were converted to United States Dollars ($) at an existing exchange rate of KES 98.00 equivalent to $1.00 in December 2018 using the following formula:

$$\text{Unit economic costs }\left(\text{\$}\right)=\frac{\text{Total economic costs}\left(\text{KES}\right)}{\text{KES }98.00}$$

This study assumed that the existing currency exchange rate of KES 98.00 in 2018 reflected the Purchasing Power Parity (PPP) globally, thus the unit economic costs were inflated to the U.S the dollar ($) Consumer Price Index (CPI) from January 2018 to December 2018 [36].

**Inflation factor and statistical uncertainties:** all the resources were categorized as recurrent costs and inflated to the U.S Dollar ($) Consumer Price Index (Calculator) for a one-year time horizon from January 2018 to December 2018 [36]. This computation adjusted the unit economic costs to purchasing power parity as a factor of inflation, and minimized statistical uncertainties due to costing data scantiness and collection methods [30,36]. The Consumer Price Index measures the mean changes in market prices over some time in which consumers pay for goods and services, thus being preferred because of being the best optimal measure of inflation [36]. Discounting for differential timing did not suffice in this study; the value of the resources was not considered for discounting (zero-rated) because of the one-year study time horizon.

**Ethical considerations:** we obtained ethical approval from Kenyatta National Hospital (KNH)-University of Nairobi (UoN) Ethics Review Committee (Ref. No: KNH-ERC/A/44). Data collected were de-identified using anonymous codes and entered in a laptop secured by an alphanumeric coded key known only to the PI to maintain confidentiality.

## Results

**Distribution of cases by category:**of the 349 cases of birth defects encountered, 340 (97.43%) were defects of the musculoskeletal system whereas 9 (2.58%) were those of the central nervous system. Of 349 cases 305 (87.39%) comprised of congenital talipes equinovarus (CTEV) consisting of unilateral congenital talipes equinovarus 300 (85.96%), bilateral congenital talipes equinovarus 3 (0.86%), unilateral congenital talipes equinovarus with germ valgus 1 (0.29%), and congenital talipes equinovarus with spina bifida 1 (0.29%). Additionally, 35 (10.03%) were cases of congenital pes planus (CPP), and 9 (2.58%) cases of neural tube defects (NTD) consisting of spina bifida 3 (0.86%), spina bifida with hydrocephalus 1 (0.29%), and hydrocephalus 5 (1.43%).

**Resource quantification for casting materials:** a set of casting materials valued at $3.9 consisting of Plaster of Paris Bandage (7.6cm×2.7m×2pcs), orthopedic cotton bandage (15cm×3m×1pc), and glycerine oil (100 mililitres×1pc) costing $1.84, $1.22, and $0.82, respectively were used to cast two cases of club foot.

**Quantification of annual inputs for 349 caseloads:** of the total unadjusted annual cost for all cases of the observed MESBDs ($392,436.49), almost two-thirds (71.48%) of resource inputs were accounted for by emoluments of occupational therapists, whereas administrative staff time accounted for about one-quarter (18%) (Table 1).

**Table 1:** quantification of annual inputs for 349 cases

Resource inputs	Item description	Quantity	Unit costs ($)	Total costs ($)	Percent (%)
Outpatient bracings	Leather foot cover, rubber sole, and a metallic rod	50 procedures	@15.31 per procedure	765.50	0.20
Outpatient tendonectomies	Orthopedic surgical procedure	14 procedures	@51.02 per procedure	714.28	0.18
Outpatient castings	Orthopedic medical procedure	1089 procedures	@1.94 per procedure	2,112.66	0.54
First and review visits for castings	First and revisits	1089 visits	@10.2 per visit	11,107.80	2.83
First and revisits all the defects	First and revisits	116 visits	@10.2 per visit	1,183.20	0.30
Estimated renting building space in square feet	898.44 (28.75×31.25) square feet @ $0.37 per hospital per month at four hospitals	898.44 @ $0.37 per month for 4 hospitals for 12 months	@$0.37×898.44×4×12	15,956.29	4.07
Emoluments	19 occupational therapists at the four study hospitals per month	19 @ $1,224.5 per month for 12 months	@$1,224.5×19×12	279,072.00	71.11
Emoluments	Four support staff at the four study hospitals	4 @ $173.5 per month for 12 months	@$173.5×4×12	8,328.00	2.12
Staff-time	Four medical superintendents at the four study hospitals	4 @ $ 20.4 per day for 24 days for 12 months	@$20.4×4×24×12	23,501.00	5.99
Staff-time	Four chief nursing officers at the four study hospitals	4 @ $10.2 per day for 24 days for a day for 12 months	@$10.2×4×24×12	11,750.50	2.99
Staff-time	Four health administrative officers at the four study hospitals	4 @ $10.2 per day for 24 days for a day for 12 months	@$10.2×4×24×12	11,750.50	2.99
Staff-time	Four orthopedic surgeons at the four study hospitals	4 @ $20.4 per day for 24 days for a day for 12 months	@$20.4×4×24×12	23,501.00	5.99
Utilities	Water and sewerage	Estimated @$ 25.51 per month for four hospitals	@$25.51×4×12	1,224.48	0.31
Utilities	Electricity	Estimated @$ 30.61 per month for four hospitals	@$30.61×4×12	1,469.28	0.37
**Total ($)**				**392,436.49**	**100.00%**

@: at, $: United States Dollar, %: percent

**Distribution of the overhead costs for specific birth defects:** the total overhead (shared) costs for these defects estimated at $376,553.05 consisted of staff time (18.72%) comprising orthopedic surgeons, medical, nursing, and administrative directors; renting building space in square feet (4.24%); emoluments of occupational therapists (74.11%); emoluments of support staff (2.21%); as well as utilities comprising water and sewerage (0.33%), and electricity (0.39%) (Table 2). The overhead costs were further allocated proportionally (percentage-based) among 305 cases of congenital talipes equinovarus (87.39%) comprised of bilateral congenital talipes equinovarus, unilateral congenital talipes equinovarus, unilateral congenital talipes equinovarus with germ valgus, and unilateral congenital talipes equinovarus with spina bifida. Congenital pes planus accounted for 35 (10.03%), whereas 9 cases of neural tube defects consisting of spina bifida, hydrocephalus, and spina bifida with hydrocephalus accounted for 2.58% of the overhead costs.

**Table 2:** distribution of overhead costs for specific birth defects

Inputs	Item descriptions	Total annual costs ($)	Percent (%)
Staff-time	Medical, orthopaedic surgeons nursing, and health administrative directors	70,503.00	18.72%
Renting building space	Estimated in square feet	15,956.29	4.24%
Emoluments	Occupational therapists	279,072.00	74.11%
Emoluments	Support staff	8,328.00	2.21%
Utilities	Water and sewerage	1,224.48	0.33%
Utilities	Electricity	1,469.28	0.39%
**Total ($)**		**376,553.05**	**100.00%**

$: United States Dollars, %: percent

**Resource inputs for the sub-groups of the defects:** of the total economic costs for the sub-groups of major external structural birth defects; congenital talipes equinovarus was estimated at $343,959.87, whereas congenital pes planus and neural tube defects were estimated at $38,322.97 and $10,153.67, respectively (Table 3).

**Table 3:** resource inputs for the sub-groups of the defects

Resource inputs for CTEV with co-defects (n=305)				
**Resource inputs**	**Item description**	**Quantity**	**Unit cost ($)**	**Annual costs ($)**
Castings	Unilateral CTEV	1028	1.94	1,994.32
	Bilateral CTEV	27	1.94	52.38
	Unilateral CTEV with germ valgus	21	1.94	40.74
	Unilateral CTEV with spina bifida	11	1.94	21.34
First and review visits	Unilateral CTEV castings	1028	10.20	10,485.60
	Bilateral CTEV castings	27	10.20	275.40
	Unilateral CTEV with germ valgus castings	21	10.20	214.20
	Unilateral CTEV with spina bifida castings	11	10.20	112.20
Bracings	Unilateral CTEV	50	15.31	765.50
Review visits	CTEV and co-defects bracings	21	10.20	214.20
Tendonectomies	Unilateral CTEV	14	51.02	714.28
Overheads	CTEV	305	1,078.92	329,069.71
**Total ($)**				**343,959.87**
**Resource inputs for congenital pes planus (n=35)**				
Castings	Congenital pes planus	2	1.94	3.90
Visits for castings	Congenital pes planus	2	10.20	20.40
Review visits	Congenital pes planus	52	10.20	530.40
Overheads	Congenital pes planus	35	1,079.09	37,768.27
**Total ($)**				**38,322.97**
**Resource inputs for neural tube defects (n=9)**				
First and review visits	Hydrocephalus	26	10.20	265.20
	Spina bifida	16	10.20	163.20
	Spina bifida with hydrocephalus	1	10.20	10.20
Overheads	Spina bifida with hydrocephalus	9	1,079.45	9,715.07
**Total ($)**				**10,153.67**

CTEV: congenital talipes equinovarus, n: sub-total number of observations, $: United States Dollar

**Estimation of unadjusted economic costs of the defects:** the total quantification of annual inputs in monetary value for 349 cases referred to as unadjusted total annual economic costs in the study were estimated at $392,436.49 (Table 1). This translated to estimated unadjusted total annual economic costs of $343,959.87 for congenital talipes equinovarus, $38,322.97 for congenital pes planus, and $10,153.67 for neural tube defects, respectively (Table 4). Thus, the estimated unadjusted unit economic costs are $1,124.46 for all the defects, $1,127.74 for congenital talipes equinovarus, $1,094.94 for congenital pes planus, and $ for neural tube defects $1,128.19 (Table 4).

**Table 4:** unadjusted and adjusted economic costs ($)

Caseloads (Morbidity data)		Unadjusted costs		Adjusted costs (inflated to CPI)	
**Type of cases**	**Frequency (n)**	**Annual total economic costs ($)**	**Unit economic costs ($)**	**Annual total economic costs ($)**	**Unit economic costs ($)**
All MESBDs	349	392,436.49	1,124.46	397,765.72	1,139.73
CTEV	305	343,959.87	1,127.74	348,630.80	1,143.05
CPP	5	38,322.97	1,094.94	38,843.39	1,109.81
NTD	9	10,153.67	1,128.19	10,291.56	1,143.51

MESBDs: major external structural birth defects; CTEV: congenital talipes equinovarus; CPP: pes planus; NTD: neural tube defects; CPI: consumer price index calculator

**Estimation of adjusted economic costs of the defects:** the unadjusted total annual economic costs estimated at $392,436.49 were adjusted for inflation factors to $397,765.72 using the consumer price index calculator (CPI) accounting for congenital talipes equinovarus at $348,630.80, congenital pes planus at $38,843.39, and neural tube defects at $10,291.56, respectively (Table 4). Thus, the adjusted unit economic costs were estimated at $1,139.73 for all types of MESBDs, $1,143.05 for CTEV, $1,109.81 for CPP, and $1,143.51 for NTD notably showing relatively similar unit economic costs of the defects (Table 4).

## Discussion

To our knowledge, this was the first study to estimate the unit economic costs of MESBDs from health care providers' economic perspective among the under-five-year-old children in Kiambu County, Kenya. Substantial public health resources are continually allocated to the healthcare systems for the care of children with MESBDs, however, the unit economic costs of care are barely known because they are rarely estimated mainly in developing countries [9,26]. Sufficient access and utilization of corrective and rehabilitative health services remain important public health interventions for improving the quality of life for birth defect-affected children globally [1-4]. Even though limited costing data, inadequate costing expertise, and the rarity of defects have been attributed to the lack of knowledge on their costs, it is of public health and economic interest to estimate the opportunity costs of health care services for MESBDs [9,26]. Worldwide, the results of this study could provide a baseline unit economic cost for the corrective and rehabilitative health services, inform the efficient allocation of health resources, stimulate, and inform costing studies, especially the costs' arms of full economic evaluation analyses [20,22,29].

The study encountered 349 cases consisting of 305 (87.39%) cases of CTEV, 35 (10.03%) cases of CPP, and nine (2.58%) cases of NTD. Congenital talipes equinovarus consisted of 300 (85.96%) cases of unilateral CTEV, three (0.86%) cases of bilateral CTEV, one (0.29%) case of unilateral CTEV with germ valgus, and one (0.29%) case of CTEV with spina bifida. Neural tube defects on the other hand comprised five (1.43%) cases of hydrocephalus, three (0.86%) cases of spina bifida, and one (0.29%) case of spina bifida with hydrocephalus. Despite variations in the number of cases (caseloads) observed for each of the defects mentioned above, this study showed a relatively similar unit economic cost for each defect in the county. The unit economic costs for NTD were approximated at $1,143.51, whereas CTEV and CPP were valued at $1,143.05, and $1,109.81, respectively. Notably, the unit economic cost of providing corrective and rehabilitative outpatient health services for these defects collectively was approximately estimated at $1,139.73.

Despite defects of the central nervous system contributing the least number (9) of cases compared to congenital talipes equinovarus (305), and congenital pes planus (35), its unit economic costs were relatively equivalent to the costs of the latter two types of the MESBDs observed in the county. Although some forms of neural tube defects are potentially fatal, the children who survive beyond infancy require substantial economic resources to deal with the related adverse health impacts [1,2,9,17]. The results of this study were indeed consistent with other research findings in the region and across the world that the greatest burden of disease associated with MESBDs is usually accounted for by the defects of the central nervous system [13,17,26]. The economic burden of spina bifida is usually substantial throughout the life of the affected individuals ascribed to the experienced high medical care expenditures in the early years of life with the defect and later reduced milestone development usually associated with spina bifida [13,37]. Our study similarly showed that neural tube defects followed by congenital pes planus accounted for the highest disease burden associated with MESBDs being shouldered by the health care systems in Kiambu county.

Even though our study estimated the economic costs of these defects among under-five-year-old children, our findings mimicked the results of other studies such as in Germany where similarly high staggering economic costs were encountered among the general population with various forms of NTD between 2006 and 2009 [13]. Worldwide, spina bifida has singly been observed to account for the highest burden of disease among other types of MESBDs [13,17]. Significant economic costs have been reported among new-born children with NTD during their first years of life, whereas, high healthcare expenditures have been observed during childhood, adolescence, and adulthood compared to the children without NTD globally [13]. In Germany, the average annual health expenditure of persons with spina bifida was estimated at €4,532, with inpatient health services contributing €1,358 (30.0%), outpatient health services €644 (14.2%), rehabilitation health services €29 (0.6%), drug therapy €562 (12.4%), and other remedies €1,939 (42.8%) [13]. In the United States among children aged between 1-17 years old, medical expenditures on spina bifida were estimated to cost 13 times as much as those on children without spina bifida [26].

Notably, annual direct economic costs of different forms of major birth defects were estimated at $2.6 billion in 2004 in the United States of America, [5,38]. Nonetheless, our study also endeavored and estimated the annual direct economic cost of MESBDs at $397,765.72. The defects encountered at the study hospitals consisted of neural tube defects, congenital talipes equinovarus, and congenital pes planus Despite different socioeconomic and demographic characteristics in Kenya and the U.S being developing, and developed countries, respectively, this was indeed a remarkable empirical effort to estimate the direct economic costs of MESBDs in Kiambu County. Costing studies were pioneered in the United States of America by Dorothy Rice in 1967, and have since been undertaken widely in Europe and Australia unlike in middle-and low-income economies [27]. Low undertakings of costing studies, particularly in low-and middle-income economies have been attributed to the scarcity of data on the burden of these defects [18,25,26]. Thus, the variations in annual direct economic costs could have been due to differences in the availability of the costing data, costing expertise, health services access, and utilization (economies of scale) [5,20,27,38]. Despite variations observed in the estimates of the economic costs, these findings point to the continuous disease burden associated with MESBDs in the county underpinning efficiency in resource utilization, and allocation for MESBDs in public and faith-based health facilities.

The few cases of NTD observed in this study could be attributed to a proportion of the carers of children with NTD seeking alternative therapies due to the associated adverse psychosocial effects experienced by the affected families [37,39-41]. Thus, the economic costs of NTD would be exponential compared to other forms of the defects observed in the study if all carers would seek care from the hospitals. Nevertheless, the estimated costs demonstrated the potential catastrophic burden of the 'silent' economic problem in the region, thus underscoring more scientific efforts to understand the magnitude of MESBDs regionally [9]. The observations made by this study have misconstrued the epidemiological and economic fallacy that MESBDs are not of public health priority relative to other health events, especially in resource-constrained countries [1-4].

**Limitations of the study:** nevertheless, some limitations were inherent in this study; first and foremost, medical records used to draw the costing data were not designed for economic evaluation studies, whereas some of the defects were likely to delay childhood milestone development prolonging the demand for corrective and rehabilitative outpatient service possibly leading to more economic expenditures. The researchers also experienced difficulties in distinguishing the extent of the cost drivers for congenital talipes equinovarus occurring with spina bifida, congenital talipes equinovarus occurring with germ valgus, and spina bifida occurring with hydrocephalus potentially inaccuracies of the profiled costing data.

## Conclusion

This study estimated the economic costs of outpatient corrective and rehabilitative health services for MESBDs in Kiambu County in Kenya. Despite the fewest caseloads for the NTD, the study showed that NTD was associated with the highest burden of disease followed by CPP in Kiambu County. Despite CTEV proportionally contributing the highest caseload for the defects, it essentially accounted for the lowest burden of the disease associated with MESBDs in the county. This observation points to adverse developmental, and psychosocial impacts among the affected children and their families not being able to access corrective and rehabilitative services. Similarly, these findings suggest a possible reduced economic productivity among the affected families arising from direct and indirect costs associated with major external structural birth defects. Therefore, we would like to recommend further studies on the direct and indirect economic costs of MESBDs among children of school-going age to understand the impacts, and the establishment of functional occupational therapy clinics in the ten sub-county hospitals to increase access to these services within Kiambu County.

### What is known about this topic

- *Major external structural birth defects are unappreciated and underprioritized as a public health problem;*
- *These defects are associated with substantial economic burdens.*

### What this study adds

- *It contributes to understanding the economic burden of birth defects regionally;*
- *Informs health planning for the defects.*
